# Robust LCEKF for Mismatched Nonlinear Systems with Non-Additive Noise/Inputs and Its Application to Robust Vehicle Navigation

**DOI:** 10.3390/s21062086

**Published:** 2021-03-16

**Authors:** Rayen Ben Abdallah, Jordi Vilà-Valls, Gaël Pagès, Damien Vivet, Eric Chaumette

**Affiliations:** Institut Supérieur de l’Aéronautique et de l’Espace (ISAE-SUPAERO), University of Toulouse, 31400 Toulouse, France; Jordi.VILA-VALLS@isae-supaero.fr (J.V.-V.); gael.pages@isae-supaero.fr (G.P.); damien.vivet@isae-supaero.fr (D.V.); eric.chaumette@isae-supaero.fr (E.C.)

**Keywords:** state estimation, linearly constrained EKF, robust filtering, model mismatch, non-additive noise, robust vehicle navigation

## Abstract

It is well known that the standard state estimation technique performance is particularly sensitive to perfect system knowledge, where the underlying assumptions are: (i) Process and measurement functions and parameters are known, (ii) inputs are known, and (iii) noise statistics are known. These are rather strong assumptions in real-life applications; therefore, a robust filtering solution must be designed to cope with model misspecifications. A possible way to design robust filters is to exploit linear constraints (LCs) within the filter formulation. In this contribution we further explore the use of LCs, derive a linearly constrained extended Kalman filter (LCEKF) for systems affected by non-additive noise and system inputs, and discuss its use for model mismatch mitigation. Numerical results for a robust tracking and navigation problem are provided to show the performance improvement of the proposed LCEKF, with respect to state-of-the-art techniques, that is, a benchmark EKF without mismatch and a misspecified EKF not accounting for the mismatch.

## 1. Introduction

The design and use of state estimation techniques is fundamental in a plethora of applications, such as robotics, tracking, guidance and navigation systems [1,2,3,4]. For a linear dynamic system, the Kalman filter (KF) is the the best linear minimum mean square error (MSE) estimator. The most widespread solution for nonlinear systems is to resort to system linearisations, leading to the so-called linearised or extended KF (EKF) [3]. In both cases, as well as for more advanced techniques such as sigma-point filters [5], the main assumption is perfect system knowledge: (i) Known process and measurement functions, and their parameters, (ii) known inputs, and (iii) known noise statistics (i.e., first and second order moments for the KF and EKF). However, these are rather strong assumptions in real-life applications, where the noise statistics’ parameters may be unknown to a certain extent, inputs may be uncertain and system parameters may be misspecified. The performance degradation of minimum MSE estimators under model mismatch has been widely reported in the literature [6,7,8,9,10], which is a reason why there exists a need to develop robust filtering techniques able to cope with mismatched systems.

Notice that in the robust filtering literature, a lot of effort has been devoted to the mitigation of possible noise statistics’ parameters misspecification, that is, to coping with non-nominal or unexpected noise behaviours. In that perspective, we may cite the robust KFs able to counteract the presence of outliers [11], the ones that estimate Gaussian noise covariances [12,13], or alternative formulations to deal with heavy-tailed distributions [14,15]. In contrast, few contributions explored how to counteract a mismatch on system matrices/functions or the filter initialisation. Within the KF framework, the latter can be solved by either the information filter form of the KF ([3] §6.2) or the so-called Fisher initialisation [16], which can be generalised by imposing initial distortionless constraints [17], i.e., the so-called minimum variance distortionless response (MVDR) estimators. In order to further generalise these MVDR results, how to incorporate non-stationary constraints within the KF has been recently proposed by Villà-Valls et al. [18], leading to a general linearly constrained KF (LCKF) formulation, which has also been shown concerning linear systems to generalise the results proposed by Teixeira et al. [19]. Indeed, the use of linear constraints (LCs) has been shown to be a promising robust filter design solution in order to mitigate the impact of misspecified system matrices [18], which complements the existing results cited above for noise parameter mismatch mitigation. Notice that other alternatives exist to estimate unknown system parameters/inputs, such as augmenting the filter state, which are not discussed in this contribution.

Once the general LCKF has been established, it is of interest to extend its use to more general nonlinear settings. A first attempt in the context of additive nonlinear systems was recently proposed by Hrustic et al. [20], where a linearly constrained EKF (LCEKF) was introduced, together with its use to mitigate parametric misspecifications on both system functions. Notice that this approach is fundamentally different from state constrained solutions [21,22], where LCs are imposed on the state and not on the filter. Overall, the use of non-stationary linear constraints for robust filtering in general nonlinear systems is still an open issue. In this contribution we further explore the use of LCs to mitigate the impact of model misspecifications in nonlinear dynamic systems with both non-additive noise and system inputs. (Notations: Italic, lower case boldface and upper case boldface indicate scalar, vector and matrix quantities; $E\left[.\right]$ denotes the expectation operator; the filter estimates based on measurements up to time *k* are denoted by ${\hat{x}}_{k|k}$; N(μ,R) is the normal distribution of mean μ and covariance R; $m_{z}$ and $C_{z}$ denote the mean and covariance of a given variable z.) The main contributions of this article are:

- The derivation of a LCEKF for general non-additive nonlinear systems.
- A demonstration of how to exploit LCs for robust filter design towards the mitigation of parametric modelling errors in both system functions.
- The LCEKF performance improvement with respect to state-of-the-art EKF solutions is validated and discussed for an illustrative tracking and navigation problem.

Notice that the proposed methodology, even if not directly stated throughout the article, can also be used to mitigate a mismatch on both process and measurement noise means (constant or time-varying), which can be regarded as inputs.

## 2. Background

Consider a linear discrete state–space model, where the state vector $x_{k}\in C^{P_{k}}$ must be estimated from the available measurements $y_{k}\in C^{N_{k}}$ (for k≥1),

(1) $$\begin{array}{cc} \; & x_{k}=F_{k-1}x_{k-1}+w_{k-1},\;y_{k}=H_{k}x_{k}+v_{k}, \end{array}$$

with $F_{k-1}\in C^{P_{k}\times P_{k-1}}$ and $H_{k}\in C^{N_{k}\times P_{k}}$ are known system model matrices, $w_{k}\in C^{P_{k}}$ and $v_{k}\in C^{N_{k}}$ are the process and measurement noise with zero mean and known covariance. If a minimum set of uncorrelation conditions holds [17], the recursive linear estimator of $x_{k}$, which minimises the MSE (for k≥1) is the KF (the superscript ${\left(\cdot \right)}^{b}$ stands for the best solution in the MSE sense),

(2) $$\begin{array}{cc} \; & {\hat{x}}_{k|k}^{b}=\left(I-K_{k}^{b}H_{k}\right)F_{k-1}{\hat{x}}_{k-1|k-1}^{b}+K_{k}^{b}y_{k}, \end{array}$$

where the optimal gain $K_{k}^{b}\in C^{P_{k}\times N_{k}}$ is the one that minimises the MSE,

(3) $$K_{k}^{b}=\arg\min_{K_{k}}\left\{P_{k|k}\left(K_{k}\right)\right\},$$

$$P_{k|k}\left(K_{k}\right)=E\left[\left({\hat{x}}_{k|k}\left(K_{k}\right)-x_{k}\right){\left({\hat{x}}_{k|k}\left(K_{k}\right)-x_{k}\right)}^{H}\right].$$

A possible way to robustify the KF is to incorporate LCs. How to use non-stationary constraints within the KF framework, leading to the so-called LCKF, has been recently proposed in [18]. In this case, the filter is

(4) $$\begin{array}{cc} {\hat{x}}_{k|k}^{b} & =\left(I-L_{k}^{b}H_{k}\right)F_{k-1}{\hat{x}}_{k-1|k-1}^{b}+L_{k}^{b}y_{k}, \end{array}$$

(5) $$\begin{array}{cc} L_{k}^{b} & =\arg\min_{L_{k}}\left\{P_{k|k}\left(L_{k}\right)\right\}\;s.t.\;L_{k}\Delta_{k}=T_{k}, \end{array}$$

computed from a “constrained” KF recursion [18], with $L_{k}^{b}\in C^{P_{k}\times N_{k}}$. Such LCKF is fully adaptive and allows to incorporatenew LCs at every time *k* ($\Delta_{k}\in C^{N_{k}\times Q_{k}}$ and $T_{k}\in C^{P_{k}\times Q_{k}}$). The use of such LCKF to robustify the filter under a mismatched model was also discussed in [18].

If we consider now an additive noise nonlinear discrete state–space model (NLDSSM),

(6) $$\begin{array}{cc} x_{k} & =f_{k-1}\left(x_{k-1}\right)+m_{w_{k-1}}+dw_{k-1}, \end{array}$$

(7) $$\begin{array}{cc} y_{k} & =h_{k}\left(x_{k}\right)+m_{v_{k}}+dv_{k}, \end{array}$$

where $f_{k-1}\left(\cdot \right)$ and $h_{k}\left(\cdot \right)$ are the known system model (process and measurement) functions, and $E\left[dw_{k-1}\right]=0$, $E\left[dv_{k}\right]=0$; a standard approach to derive a nonlinear filter of $x_{k}$ is to assume that Equations (6) and (7) can be linearised at the vicinity of a nominal trajectory [3] yielding the standard EKF,

(8) $$\begin{array}{cc} {\hat{x}}_{k|k}^{b} & ={\hat{x}}_{k|k-1}^{b}+K_{k}^{b}\left(y_{k}-{\hat{y}}_{k|k-1}^{b}\right), \end{array}$$

(9) $$\begin{array}{cc} {\hat{x}}_{k|k-1}^{b} & ≃f_{k-1}\left({\hat{x}}_{k-1|k-1}^{b}\right)+m_{w_{k-1}}, \end{array}$$

(10) $$\begin{array}{cc} {\hat{y}}_{k|k-1}^{b} & ≃h_{k}\left({\hat{x}}_{k|k-1}^{b}\right)+m_{v_{k}}, \end{array}$$

where $K_{k}^{b}$ is computed as in the unconstrained KF with $F_{k-1}≃\frac{\partial f_{k-1}\left({\hat{x}}_{k-1|k-1}^{b}\right)}{\partial x_{k-1}^{T}},H_{k}≃\frac{\partial h_{k}\left({\hat{x}}_{k|k-1}^{b}\right)}{\partial x_{k}^{T}}$. As has been recently proposed in [20], the corresponding LCEKF for additive noise systems is ${\hat{x}}_{k|k}^{b}={\hat{x}}_{k|k-1}^{b}+L_{k}^{b}\left(y_{k}-{\hat{y}}_{k|k-1}^{b}\right)$, where the constrained gain $L_{k}^{b}$ is computed as in the original LCKF but using the linearised matrices, as done in the EKF. Notice that the LCKF in [18] is not applicable to nonlinear systems, and the LCEKF in [20] is only valid for additive systems. The goal of this article is to further analyse these methods and extend their use to more general settings.

## 3. An LCEKF with Non-Additive Noise and System Inputs, and Its Use in Robust Filtering

We consider now a more general NLDSSM, represented by the following state and measurement equations (k≥1)

(11) $$\left\{\begin{array}{c} x_{k}=f_{k-1}\left(x_{k-1},w_{k-1}\right)\\ y_{k}=h_{k}\left(x_{k},v_{k}\right) \end{array}\right.,x_{k}\in C^{P_{k}},y_{k}\in C^{N_{k}},$$

with $f_{k-1}\left(\cdot \right)$ and $h_{k}\left(\cdot \right)$ being known system model functions, and both noises $w_{k}$ and $v_{k}$ with known mean and covariance. First, we want to obtain the EKF-type linear MMSE filter of $x_{k}$ that can be written as in the previous cases. If $tr\left(C_{x_{0}}\right)\ll 1$, $tr\left(C_{w_{k-1}}\right)\ll 1$, $tr\left(C_{x_{k-1}}\right)\ll 1$ and $tr\left(C_{v_{k}}\right)\ll 1$, we can resort to a first order Taylor expansion of $f_{k-1}\left(x_{k-1},w_{k-1}\right)$ at the vicinity of $\left(m_{x_{k-1}},m_{w_{k-1}}\right)$, and of $h_{k}\left(x_{k},v_{k}\right)$ at the vicinity of $\left(m_{x_{k}},m_{v_{k}}\right)$. In such a case, if we restrict ourselves, for legibility, to the usual uncorrelation conditions, i.e., $C_{x_{0},w_{k}}=0$, $C_{x_{0},v_{k}}=0$, $C_{w_{l},w_{k}}=C_{w_{k}}\delta_{k}^{l}$, $C_{v_{l},v_{k}}=C_{v_{k}}\delta_{k}^{l}$, $C_{w_{l},v_{k}}=0$, the general form of the EKF becomes

(12) $$\begin{array}{cc} \; & {\hat{x}}_{k|k}^{b}={\hat{x}}_{k|k-1}^{b}+K_{k}^{b}\left(y_{k}-{\hat{y}}_{k|k-1}^{b}\right), \end{array}$$

(13) $$\begin{array}{cc} \; & {\hat{x}}_{k|k-1}^{b}≃f_{k-1}\left({\hat{x}}_{k-1|k-1}^{b},m_{w_{k-1}}\right), \end{array}$$

(14) $${\hat{y}}_{k|k-1}^{b}≃h_{k}\left({\hat{x}}_{k|k-1}^{b},m_{v_{k}}\right),$$

$$\left\{ \begin{array}{c} P_{k|k-1}^{b}=F_{k-1}P_{k-1|k-1}^{b}F_{k-1}^{H}+N_{k-1}C_{w_{k-1}}N_{k-1}^{H},\\ S_{k|k-1}^{b}=H_{k}P_{k|k-1}^{b}H_{k}^{H}+M_{k}C_{v_{k}}M_{k}^{H},\\ K_{k}^{b}=P_{k|k-1}^{b}H_{k}^{H}{\left(S_{k|k-1}^{b}\right)}^{-1},\\ P_{k|k}^{b}=\left(I-K_{k}^{b}H_{k}\right)P_{k|k-1}^{b}, \end{array}\right.$$

with $F_{k-1}$, $N_{k-1}$, $H_{k}$, $M_{k}$ approximated as: $F_{k-1}≃\frac{\partial \left({\hat{x}}_{k-1|k-1}^{b},m_{w_{k-1}}\right)}{\partial x_{k-1}^{T}}$, $N_{k-1}≃\frac{\partial f_{k-1}\left({\hat{x}}_{k-1|k-1}^{b},m_{w_{k-1}}\right)}{\partial w_{k-1}^{T}}$, $H_{k}≃\frac{\partial h_{k}\left({\hat{x}}_{k|k-1}^{b},m_{v_{k}}\right)}{\partial x_{k}^{T}}$ and $M_{k}≃\frac{\partial h_{k}\left({\hat{x}}_{k|k-1}^{b},m_{v_{k}}\right)}{\partial v_{k}^{T}}$. Notice that we do not explicitly exhibit the dependence on the system inputs in the previous EKF, which can be embedded in the noise means $m_{w_{k-1}}$ and $m_{v_{k}}$.

### 3.1. Mismatched and True System Models

As previously stated, it is unlikely that the practitioner has a full knowledge of the system, and therefore we want to cope with the situation where there is a true $M_{T}$ and a mismatched/assumed $M_{A}$ NLDSSM as follows,

(15) $$\begin{array}{cc} \; & M_{A}:\left\{\begin{array}{c} x_{k}^{\prime }=f_{k-1}\left(x_{k-1}^{\prime },m_{w_{k-1}}+dw_{k-1},\hat{!}\right)\\ y_{k}=h_{k}\left(x_{k}^{\prime },m_{v_{k}}+dv_{k},\hat{‘}\right) \end{array}\right. \end{array}$$

(16) $$\begin{array}{cc} \; & M_{T}:\left\{\begin{array}{c} x_{k}=f_{k-1}\left(x_{k-1},m_{w_{k-1}}+dw_{k-1},!\right)\\ y_{k}=h_{k}\left(x_{k},m_{v_{k}}+dv_{k},‘\right) \end{array}\right. \end{array}$$

with $w_{k-1}=m_{w_{k-1}}+dw_{k-1}$, $v_{k}=m_{v_{k}}+dv_{k}$, $E\left[dw_{k-1}\right]=0$ and $E\left[dv_{k}\right]=0$. Since the EKF of $x_{k}$ is based on the measurements and our knowledge of the model dynamics, any mismatch between the true model dynamics $M_{T}$ and the assumed one $M_{A}$ leads to a suboptimal filter, and possibly to a filter with bad performance, as the discrepancy between the two models increases.

The existence of uncertainty on system nonlinear functions, either because of a parametric model error or a mismatch on system inputs, is taken into account as $f_{k-1}\left(.\right)≜f_{k-1}\left(.,!\right)$ and $h_{k}\left(.\right)≜h_{k}\left(.,‘\right)$, where ! and ‘ are deterministic vector values or system inputs, and the possible parametric model mismatch is given by $\hat{!}=!+d\hat{!}$ and $\hat{‘}=‘+d\hat{‘}$. If errors $d\hat{!}$ and $d\hat{‘}$ are small, then the true state and measurement nonlinear functions differ from the assumed ones via first order Taylor series as follows,

(17) $$f_{k-1}\left(x_{k-1},w_{k-1},!\right)-f_{k-1}\left(x_{k-1},w_{k-1},\hat{!}\right)≃\frac{\partial f_{k-1}\left(x_{k-1},w_{k-1},\hat{!}\right)}{\partial {!}^{T}}\left(!-\hat{!}\right),$$

$$h_{k}\left(x_{k},v_{k},‘\right)-h_{k}\left(x_{k},v_{k},\hat{‘}\right)≃\frac{\partial h_{k}\left(x_{k},v_{k},\hat{‘}\right)}{\partial {‘}^{T}}\left(‘-\hat{‘}\right).$$

At time k≥1, the EKF of $x_{k}$ is obtained from the Kalman-like recursion Equation (12) computed with the assumed model $M_{A}$,

(18) $$\begin{array}{cc} \; & {\hat{x}}_{k|k}\left(L_{k}\right)={\hat{x}}_{k|k-1}^{b}+L_{k}\left(y_{k}-{\hat{y}}_{k|k-1}^{b}\right), \end{array}$$

(19) $$\begin{array}{cc} \; & {\hat{x}}_{k|k-1}^{b}=f_{k-1}\left({\hat{x}}_{k-1|k-1}^{b},m_{w_{k-1}},\hat{!}\right), \end{array}$$

(20) $$\begin{array}{cc} \; & {\hat{y}}_{k|k-1}^{b}=h_{k}\left({\hat{x}}_{k|k-1}^{b},m_{v_{k}},\hat{‘}\right), \end{array}$$

with the gain $L_{k}$ obtained from the MSE minimisation, and also computed with $M_{A}$.

### 3.2. Impact of System Parametric Modelling Errors

The first step towards the system model mismatch mitigation is to compute the estimation error induced by the use of the assumed model $M_{A}$. Among the possible estimation error breakdowns, we look for the one that makes the terms in (17) appear, and which reduces to the error analysed in [18] when the system becomes linear (as shown in Section 3.5). An iterative approach was taken to integrate these two requirements and led to breaking down the error as

(21) $$\begin{array}{cc} & {\hat{x}}_{k|k}\left(L_{k}\right)-x_{k}={\hat{x}}_{k|k-1}^{b}-x_{k}+L_{k}\left(y_{k}-{\hat{y}}_{k|k-1}^{b}\right)\\ & =f_{k-1}\left({\hat{x}}_{k-1|k-1}^{b},m_{w_{k-1}},\hat{!}\right)-f_{k-1}\left(x_{k-1},m_{w_{k-1}}+dw_{k-1},\hat{!}\right)\\ & +L_{k}h_{k}\left(f_{k-1}\left(x_{k-1},m_{w_{k-1}}+dw_{k-1},\hat{!}\right),m_{v_{k}}+dv_{k},\hat{‘}\right)\\ \; & -L_{k}h_{k}\left(f_{k-1}\left({\hat{x}}_{k-1|k-1}^{b},m_{w_{k-1}},\hat{!}\right),m_{v_{k}},\hat{‘}\right)+{”}_{k}\left(L_{k}\right), \end{array}$$

with the additional error term,

(22) $$\begin{array}{cc} & {”}_{k}\left(L_{k}\right)=f_{k-1}\left(x_{k-1},w_{k-1},\hat{!}\right)-f_{k-1}\left(x_{k-1},w_{k-1},!\right)\\ \; & +L_{k}\left(h_{k}\left(f_{k-1}\left(x_{k-1},w_{k-1},!\right),v_{k},‘\right)-h_{k}\left(f_{k-1}\left(x_{k-1},w_{k-1},\hat{!}\right),v_{k},\hat{‘}\right)\right). \end{array}$$

Indeed, if we assume that ${\hat{x}}_{k-1|k-1}^{b}$ is a good enough unbiased estimate of $x_{k-1}$, that is,

(23) $$\begin{array}{cc} \; & E\left[{\hat{x}}_{k-1|k-1}^{b}\right]=E\left[x_{k-1}\right]=m_{x_{k-1}} \end{array}$$

(24) $$\begin{array}{cc} \; & tr\left(P_{k-1|k-1}^{b}\right)=E[||{\hat{x}}_{k-1|k-1}^{b}-x_{k-1}{\left|\right|}^{2}]\ll 1, \end{array}$$

then the first order approximations of Equations (21) and (22) are

(25) $$\begin{array}{cc} \; & {\hat{x}}_{k|k}\left(L_{k}\right)-x_{k}≃\left(I-L_{k}{\hat{H}}_{k}\right)\left({\hat{F}}_{k-1}\left({\hat{x}}_{k-1|k-1}^{b}-x_{k-1}\right)-{\hat{N}}_{k-1}dw_{k-1}\right)+L_{k}{\hat{M}}_{k}dv_{k}+{”}_{k}\left(L_{k}\right), \end{array}$$

(26) $$\begin{array}{cc} \; & {”}_{k}\left(L_{k}\right)≃L_{k}\frac{\partial h_{k}\left(x_{k},v_{k},\hat{‘}\right)}{\partial {‘}^{T}}\left(‘-\hat{‘}\right)-\left(I-L_{k}{\hat{H}}_{k}\right)\frac{\partial f_{k-1}\left(x_{k-1},w_{k-1},\hat{!}\right)}{\partial {!}^{T}}\left(!-\hat{!}\right), \end{array}$$

where the linearised matrices for the assumed model are now ${\hat{F}}_{k}=\frac{\partial f_{k}\left(m_{x_{k}},m_{w_{k}},\hat{!}\right)}{\partial x_{k}^{T}},{\hat{N}}_{k}=\frac{\partial f_{k}\left(m_{x_{k}},m_{w_{k}},\hat{!}\right)}{\partial w_{k}^{T}}$, ${\hat{H}}_{k}=\frac{\partial h_{k}\left({\hat{m}}_{x_{k}},m_{v_{k}},\hat{‘}\right)}{\partial x_{k}^{T}},{\hat{M}}_{k}=\frac{\partial h_{k}\left({\hat{m}}_{x_{k}},m_{v_{k}},\hat{‘}\right)}{\partial v_{k}^{T}}$, with mean terms given by $m_{x_{k}}≃f_{k-1}\left(m_{x_{k-1}},m_{w_{k-1}},!\right)$ and ${\hat{m}}_{x_{k}}≃f_{k-1}\left(m_{x_{k-1}},m_{w_{k-1}},\hat{!}\right)$.

### 3.3. Mitigation of Parametric Modelling Errors through LCs

Once we have established the additional error induced by the model mismatch, the practitioner needs an efficient way to mitigate it. Notice that

(27) $$E\left[{\hat{x}}_{k-1|k-1}^{b}-x_{k-1}\right]=0\Rightarrow \;E\left[{\hat{x}}_{k|k}\left(L_{k}\right)-x_{k}\right]=E\left[{”}_{k}\left(L_{k}\right)\right],$$

$$E\left[{”}_{k}\left(L_{k}\right)\right]≃L_{k}E\left[\frac{\partial h_{k}\left(x_{k},v_{k},\hat{‘}\right)}{\partial {‘}^{T}}\right]\left(‘-\hat{‘}\right)-\left(I-L_{k}{\hat{H}}_{k}\right)E\left[\frac{\partial f_{k-1}\left(x_{k-1},w_{k-1},\hat{!}\right)}{\partial {!}^{T}}\right]\left(!-\hat{!}\right),$$

therefore, we can conclude that

(28) $$\begin{array}{cc} \; & \forall \left(!-\hat{!}\right),\forall (\left(‘-\hat{‘}\right),\;E\left[{”}_{k}\left(L_{k}\right)\right]=0\;\Leftrightarrow \left\{\begin{array}{c} L_{k}E\left[\frac{\partial h_{k}\left(x_{k},v_{k},\hat{‘}\right)}{\partial {‘}^{T}}\right]=0\\ \left(I-L_{k}{\hat{H}}_{k}\right)E\left[\frac{\partial f_{k-1}\left(x_{k-1},w_{k-1},\hat{!}\right)}{\partial {!}^{T}}\right]=0 \end{array}\right., \end{array}$$

which defines a sensible set of constraints in order to mitigate at first order, which mainly consists of the bias, the error introduced by parametric modelling errors in the nonlinear system functions. However, expectations are probably not computable in several applications, and

(29) $$\begin{array}{c} dH_{k}≜E\left[\frac{\partial h_{k}\left(x_{k},v_{k},\hat{‘}\right)}{\partial {‘}^{T}}\right]≃\frac{\partial h_{k}\left({\hat{x}}_{k|k-1}^{b},m_{v_{k}},\hat{‘}\right)}{\partial {‘}^{T}},\\ dF_{k}≜E\left[\frac{\partial f_{k}\left(x_{k},w_{k},\hat{!}\right)}{\partial {!}^{T}}\right]≃\frac{\partial f_{k}\left({\hat{x}}_{k|k}^{b},m_{w_{k}},\hat{!}\right)}{\partial {!}^{T}}. \end{array}$$

These LCs provide a non degenerate solution [18] only if $rank\left(dF_{k-1}\right)=R_{k-1}<P_{k-1}$. Then, Equation (28) can be recast as $\left\{L_{k}dH_{k}=0,\;L_{k}\left({\hat{H}}_{k}dF_{k-1}\right)=dF_{k-1}\right\}$. Let $dF_{k-1}=U_{k-1}d\Phi_{k-1}$ be the singular value decomposition (SVD) of $dF_{k-1}$, where $U_{k-1}\in C^{P_{k}\times R_{k}}$ has full rank $R_{k}<P_{k}$ and $d\Phi_{k-1}\in C^{R_{k}\times P_{k-1}}$. Then Equation (28) becomes,

(30) $$\{L_{k}dH_{k}=0,\;L_{k}\left({\hat{H}}_{k}U_{k-1}\right)=U_{k-1}\}.$$

### 3.4. Exploiting LCs for Robust Filtering under Mismatch

Notice that by imposing LCs in Equation (30), the estimate obtained with the mismatched NLDSSM Equation (15) is matched to the true observation in Equation (16). Indeed, the LCKF minimises the MSE associated to the true state $x_{k}$, matching the true observations to the assumed model. We detail the robust LCEKF methodology in the sequel. At every time *k*,

*Prediction*

(31) $${\hat{x}}_{k|k-1}^{b}≃f_{k-1}\left({\hat{x}}_{k-1|k-1}^{b},m_{w_{k-1}},\hat{!}\right),$$

$$P_{k|k-1}^{b}={\hat{F}}_{k-1}P_{k-1|k-1}^{b}{\hat{F}}_{k-1}^{H}+{\hat{N}}_{k-1}C_{w_{k-1}}{\hat{N}}_{k-1}^{H},$$

(32) $${\hat{y}}_{k|k-1}^{b}≃h_{k}\left({\hat{x}}_{k|k-1}^{b},m_{v_{k}},\hat{‘}\right),$$

$$S_{k|k-1}^{b}={\hat{H}}_{k}P_{k|k-1}^{b}{\hat{H}}_{k}^{H}+{\hat{M}}_{k}C_{v_{k}}{\hat{M}}_{k}^{H},$$

*Unconstrained Gain*

(33) $$K_{k}=P_{k|k-1}^{b}{\hat{H}}_{k}^{H}{\left(S_{k|k-1}^{b}\right)}^{-1},$$

*Linear Constraints*

$$\Delta_{k}=\left[dH_{k}\;\;{\hat{H}}_{k}U_{k-1}\right],\;T_{k}=\left[0\;\;U_{k-1}\right]$$

$$\Gamma_{k}=T_{k}-K_{k}\Delta_{k},\;\Psi_{k}=\Delta_{k}^{H}{\left(S_{k|k-1}^{b}\right)}^{-1}\Delta_{k}$$

*Constrained Gain*

(34) $$L_{k}^{b}=K_{k}+\Gamma_{k}\Psi_{k}^{-1}\Delta_{k}^{H}{\left(S_{k|k-1}^{b}\right)}^{-1}$$

*Update*

(35) $${\hat{x}}_{k|k}^{b}={\hat{x}}_{k|k-1}^{b}+L_{k}^{b}\left(y_{k}-{\hat{y}}_{k|k-1}^{b}\right),$$

$$P_{k|k}^{b}=\left(I-K_{k}{\hat{H}}_{k}\right)P_{k|k-1}^{b}+\Gamma_{k}\Psi_{k}^{-1}\Gamma_{k}^{H}$$

with the matrices ${\hat{F}}_{k-1}$, ${\hat{N}}_{k-1}$, ${\hat{H}}_{k}$, ${\hat{M}}_{k}$ evaluated at ${\hat{x}}_{k-1|k-1}^{b}$ and ${\hat{x}}_{k|k-1}^{b}$ instead of at the unknown state means.

### 3.5. Special Cases: Linear and Additive Systems

If we assume that the NLDSSMs Equations (15) and (16) become linear, we have that,

(36) $$\begin{array}{cc} f_{k-1}\left(x_{k-1},w_{k-1},!\right) & =F_{k-1}\left(!\right)x_{k-1}+w_{k-1}, \end{array}$$

(37) $$\begin{array}{cc} h_{k}\left(x_{k},v_{k},‘\right) & =H_{k}\left(‘\right)x_{k}+v_{k}, \end{array}$$

with $F_{k-1}\left(!\right)=\left[f_{k-1}^{1}\left(!\right)\ldots f_{k-1}^{P_{k-1}}\left(!\right)\right]$ and $H_{k}\left(‘\right)=\left[h_{k}^{1}\left(‘\right)\ldots h_{k}^{P_{k}}\left(‘\right)\right]$, leading to ${\hat{F}}_{k-1}=F_{k-1}\left(\hat{!}\right)$, ${\hat{N}}_{k-1}=I$, ${\hat{H}}_{k}=H_{k}\left(\hat{‘}\right)$ and ${\hat{M}}_{k}=I$. Moreover, in this case,

(38) $$\frac{\partial f_{k-1}\left(x_{k-1},w_{k-1},\hat{!}\right)}{\partial {!}^{T}}\left(!-\hat{!}\right)≃\left(F_{k-1}\left(!\right)-F_{k-1}\left(\hat{!}\right)\right)x_{k-1}=dF_{k-1}x_{k-1},$$

$$\frac{\partial h_{k}\left(x_{k},v_{k},\hat{‘}\right)}{\partial {‘}^{T}}\left(‘-\hat{‘}\right)≃\left(H_{k}\left(‘\right)-H_{k}\left(\hat{‘}\right)\right)x_{k}=dH_{k}x_{k},$$

then, Equation (26) reduces to the linear system error analysed in [18], which confirms the relevance of Equations (25) and (26) as a first order approximation of Equations (21) and (22) and the choice of the form of Equation (21).

If we assume the intermediate case where the NLDSSMs Equations (15) and (16) are still nonlinear but with additive noise, then,

(39) $$\begin{array}{cc} f_{k-1}\left(x_{k-1},w_{k-1},!\right) & =f_{k-1}\left(x_{k-1},!\right)+w_{k-1}, \end{array}$$

(40) $$\begin{array}{cc} h_{k}\left(x_{k},v_{k},‘\right) & =h_{k}\left(x_{k},‘\right)+v_{k}, \end{array}$$

leading to ${\hat{F}}_{k}=\frac{\partial f_{k}\left(m_{x_{k}},\hat{!}\right)}{\partial x_{k}^{T}}$, ${\hat{N}}_{k}=I$, ${\hat{H}}_{k}=\frac{\partial h_{k}\left({\hat{m}}_{x_{k}},\hat{‘}\right)}{\partial x_{k}^{T}}$ and ${\hat{M}}_{k}=I$, with $m_{x_{k}}≃f_{k-1}\left(m_{x_{k-1}},!\right)$ and ${\hat{m}}_{x_{k}}≃f_{k-1}\left(m_{x_{k-1}},\hat{!}\right)$. Because the noise terms do not depend on the system parameters or inputs, we simply have that,

$$\begin{array}{cc} & \frac{\partial f_{k-1}\left(x_{k-1},w_{k-1},\hat{!}\right)}{\partial {!}^{T}}=\frac{\partial f_{k-1}\left(x_{k-1},\hat{!}\right)}{\partial {!}^{T}},\\ & \frac{\partial h_{k}\left(x_{k},v_{k},\hat{‘}\right)}{\partial {‘}^{T}}=\frac{\partial h_{k}\left(x_{k},\hat{‘}\right)}{\partial {‘}^{T}}. \end{array}$$

Finally, if we consider nonlinear additive inputs of the form, $f_{k-1}\left(x_{k-1},w_{k-1},!\right)=f_{k-1}\left(x_{k-1}\right)+g_{k-1}\left(!\right)+w_{k-1}$, then $\partial f_{k-1}\left(x_{k-1},w_{k-1},\hat{!}\right)/\partial {!}^{T}=\partial g_{k-1}\left(\hat{!}\right)/\partial {!}^{T}$ and we recover the results in [20].

## 4. Illustrative Example: Robust Vehicle Tracking and Navigation

**Figure 1:**
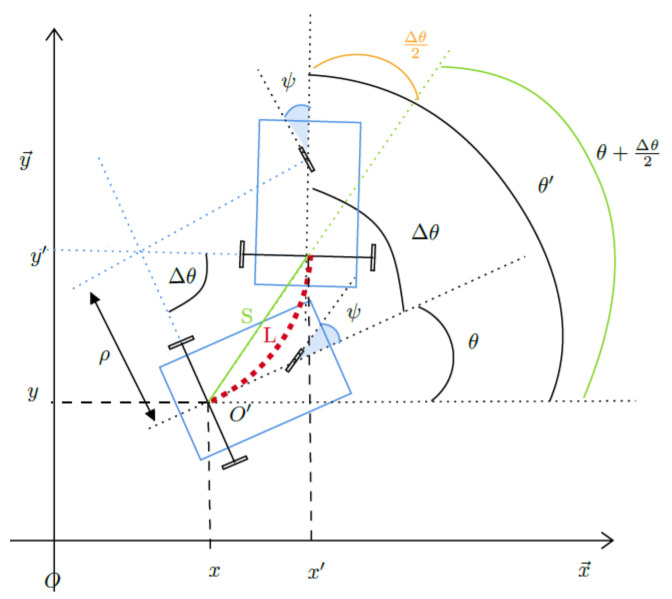
Three-wheeled vehicle model in movement.

To illustrate the validity of the proposed LCEKF and its performance improvement with respect to standard (not robust) EKF solutions, we analyse a two vehicle tracking and navigation problem. We consider for both vehicles a tricycle dynamic model (i.e., two rear wheels provide the speed, and the front one controls the direction), also known as the Ackerman model [23], which is used to describe the kinematic behavior (2D position and orientation) of most parts of vehicles with three and four wheels (see Figure 1). In the sequel subscript ${\left(\cdot \right)}_{T}$ refers to the tracking vehicle, and ${\left(\cdot \right)}_{B}$ to the tracked one. Refer to Appendix A for details.

**Figure 2:**
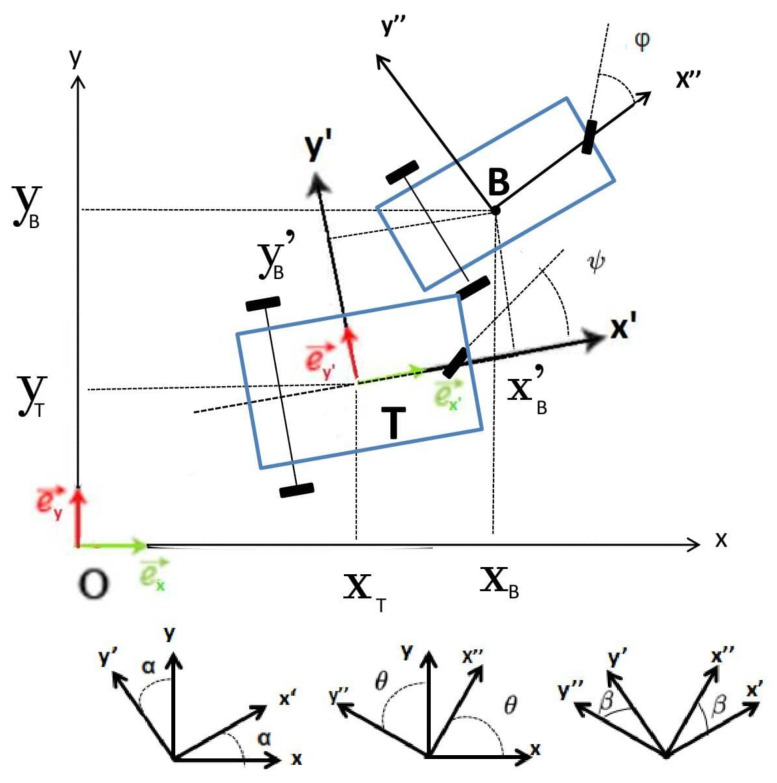
Two vehicle tracker (T) and tracked (B) scenario, and the different vehicle coordinate systems.

As shown in Figure 2, we consider a tracking vehicle at $\left(x_{T},y_{T}\right)$ with linear velocity $V_{T}$, orientation α and steering angle ψ, and a tracked vehicle at $\left(x_{B},y_{B}\right)$ with linear velocity $V_{B}$, orientation θ and steering angle ϕ. The state at discrete time *k* is $x_{k}^{T}=\left[{\left(x_{B}\right)}_{k},{\left(y_{B}\right)}_{k},\theta_{k},{\left(x_{T}\right)}_{k},{\left(y_{T}\right)}_{k},\alpha_{k}\right]$ (in the global frame (xOy)), to be estimated from measurements $y_{k}^{T}=\left[{\left(x_{B}^{\prime }\right)}_{k},{\left(y_{B}^{\prime }\right)}_{k},\beta_{k},{\left(x_{T}\right)}_{k},{\left(y_{T}\right)}_{k},\alpha_{k}\right]$, where $\left({\left(x_{B}^{\prime }\right)}_{k},{\left(y_{B}^{\prime }\right)}_{k}\right)$ and $\beta_{k}$ are the tracked vehicle position and orientation in the tracker (x’O’y’) coordinate frame. Velocities, $V_{T}$ and $V_{B}$, and steering information, ψ and ϕ, are obtained from an odometer and steering angle sensor. We consider a cooperative tracking process where the tracked vehicle communicates its navigation information, i.e., $V_{B}$ and ϕ, to the tracking vehicle. Therefore, we have an input parameter vector $u^{T}=\left[V_{B},\phi ,D_{B},V_{T},\psi ,D_{T}\right]$, with $D_{B}$ and $D_{T}$ being the distance from the front wheel to the rear axle.

### 4.1. State Model with Both Additive and Non-Additive Noise

We consider a nonlinear state model of the form $x_{k}=f_{k-1}\left(x_{k-1},u,w_{k-1}\right)+w_{k-1}^{\prime }$. The additive noise $w_{k-1}^{\prime }∼N\left(m_{w}^{\prime },C_{w}^{\prime }\right)$ models deviations on the vehicles’ position and orientation, due to wind perturbations or unmodeled accelerations, with $C_{w}^{\prime }=\mathrm{diag}\left(\sigma_{x_{B}}^{2},\sigma_{y_{B}}^{2},\sigma_{\theta }^{2},\sigma_{x_{T}}^{2},\sigma_{y_{T}}^{2},\sigma_{\alpha }^{2}\right)$. The non-additive noise $w_{k-1}={\left[w_{v},w_{\psi }\right]}_{k-1}^{T}∼N\left(m_{w},C_{w}\right)$ models perturbations on the input parameters $V_{T}$ and ψ (i.e., the odometer may be affected by tire pressure and road conditions, and the steering information is subject to noise due to friction and wind), with $C_{w}=\mathrm{diag}\left(\sigma_{v}^{2},\sigma_{\psi }^{2}\right)$,

(41) $$\begin{array}{cc} \; & x_{k}=x_{k-1}+g_{k-1}\left(x_{k-1},u,w_{k-1}\right)+w_{k-1}^{\prime }, \end{array}$$

(42) $$\begin{array}{cc} \; & {\left[g_{k-1}\left(\cdot \right)\right]}_{1}=\frac{2D_{B}}{\tan(\phi )}\;\sin\left(\frac{V_{B}\;dt\;\tan\left(\phi \right)}{2D_{B}}\right)\cos\left(\theta_{k-1}+\frac{V_{B}\;dt\;\tan\left(\phi \right)}{2D_{B}}\right), \end{array}$$

(43) $$\begin{array}{cc} \; & {\left[g_{k-1}\left(\cdot \right)\right]}_{2}=\frac{2D_{B}}{\tan(\phi )}\;\sin\left(\frac{V_{B}\;dt\;\tan\left(\phi \right)}{2D_{B}}\right)\sin\left(\theta_{k-1}+\frac{V_{B}\;dt\;\tan\left(\phi \right)}{2D_{B}}\right), \end{array}$$

(44) $$\begin{array}{cc} \; & {\left[g_{k-1}\left(\cdot \right)\right]}_{3}=\frac{V_{B}\;dt}{D_{B}}\tan\left(\phi \right), \end{array}$$

(45) $$\begin{array}{cc} \; & {\left[g_{k-1}\left(\cdot \right)\right]}_{4}=\frac{2D_{T}}{\tan(\hat{\psi })}\;\sin\left(\frac{{\hat{V}}_{T}\;dt\;\tan\left(\hat{\psi }\right)}{2D_{T}}\right)\cos\left(\alpha_{k-1}+\frac{{\hat{V}}_{T}\;dt\;\tan\left(\hat{\psi }\right)}{2D_{T}}\right), \end{array}$$

(46) $$\begin{array}{cc} \; & {\left[g_{k-1}\left(\cdot \right)\right]}_{5}=\frac{2D_{T}}{\tan(\hat{\psi })}\;\sin\left(\frac{{\hat{V}}_{T}\;dt\;\tan\left(\hat{\psi }\right)}{2D_{T}}\right)\sin\left(\alpha_{k-1}+\frac{{\hat{V}}_{T}\;dt\;\tan\left(\hat{\psi }\right)}{2D_{T}}\right), \end{array}$$

(47) $$\begin{array}{cc} \; & {\left[g_{k-1}\left(\cdot \right)\right]}_{6}=\frac{{\hat{V}}_{T}\;dt}{D_{T}}\tan\left(\hat{\psi }\right), \end{array}$$

with ${\hat{V}}_{T}=V_{T}+w_{v}$ and $\hat{\psi }=\psi +w_{\psi }$.

### 4.2. Nonlinear Measurement Model and Mismatch

The measurement principle is illustrated in Figure 2, where the tracker locates itself at point $O^{\prime }$ and the tracked vehicle at point *B* in its coordinate frame (x’O’y’),

(48) $$\begin{array}{cc} \; & y_{k}=h_{k}\left(x_{k}\right)+n_{k},\;n_{k}∼N\left(0,C_{n}\right), \end{array}$$

(49) $$\begin{array}{cc} \; & C_{n}=\mathrm{diag}\left(\sigma_{x^{\prime }}^{2},\sigma_{y^{\prime }}^{2},\sigma_{\beta }^{2},\sigma_{x_{T}^{\prime }}^{2},\sigma_{y_{T}^{\prime }}^{2},\sigma_{\alpha^{\prime }}^{2}\right), \end{array}$$

and $h_{k}\left(x_{k}\right)$ is given by

(50) $$\begin{array}{c} h_{k}\left(x_{k}\right)=\left[\begin{array}{cc} \begin{array}{c} \left[\begin{array}{cc} \cos(-\alpha_{k}) & -\sin(-\alpha_{k})\\ \sin(-\alpha_{k}) & \cos(-\alpha_{k}) \end{array}\right] \end{array}\; & O\\ O & I_{4} \end{array}\right]\left[\begin{array}{c} \begin{array}{c} {\left(x_{T}\right)}_{k}-{\left(x_{B}\right)}_{k}\\ {\left(y_{T}\right)}_{k}-{\left(y_{B}\right)}_{k}\\ \theta_{k}-\alpha_{k}\\ {\left(x_{T}\right)}_{k}\\ {\left(y_{T}\right)}_{k}\\ \alpha_{k} \end{array} \end{array}\right]. \end{array}$$

We consider the case where we may have an imperfect knowledge on the system inputs. In practice, the distance $D_{B}$ and $D_{T}$ are not accurate, i.e., there exists a deviation between the true values, $D_{B}$ and $D_{T}$, and the assumed ones, ${\hat{D}}_{B}$ and ${\hat{D}}_{T}$, $dD_{B}=D_{B}-{\hat{D}}_{B}$ and $dD_{T}=D_{T}-{\hat{D}}_{T}$. In order to mitigate the possible impact of such error, we resort to the LCEKF in Section 3.4, incorporating the constraint

(51) $$\left(I-L_{k}{\hat{H}}_{k}\right)dF_{k-1}=0,\;{\left.dF_{k}=\frac{\partial f_{k}\left(x_{k},u,w_{k}\right)}{\partial a^{T}}\right|}_{x_{k}={\hat{x}}_{k|k},u=\hat{u},w_{k}=m_{w}},$$

with the input vector given by $\hat{u}={\left[V_{B},\phi ,{\hat{D}}_{B},V_{T},\psi ,{\hat{D}}_{T}\right]}^{T}$ in the additive noise case, or $\hat{u}={\left[V_{B},\phi ,{\hat{D}}_{B},{\hat{V}}_{T},\hat{\psi },{\hat{D}}_{T}\right]}^{T}$ in the non-additive noise one, and $a={\left[D_{B},D_{T}\right]}^{T}$.

### 4.3. Scenarios

We consider the following setup: dt=1 ms, $C_{x_{0}}=0$, $x_{0}={\left(10,20,1^{\circ },8,19,1.5^{\circ }\right)}^{T}$, and a measurement noise with $\sigma_{x^{\prime }}=1$ m, $\sigma_{y^{\prime }}=1$ m, $\sigma_{\beta }=0.1^{\circ }$, $\sigma_{x_{T}^{\prime }}=1$ m, $\sigma_{y_{T}^{\prime }}=1$ m and $\sigma_{\alpha^{\prime }}=0.1^{\circ }$. Three scenarios are of interest:

- S1) Only additive noise ($w_{k-1}=0$): $m_{w}^{\prime }=0$, $\sigma_{x_{B}}=\sigma_{y_{B}}=\sigma_{x_{T}}=\sigma_{y_{T}}=0.1$ m and $\sigma_{\theta }=\sigma_{\alpha }=0.1^{\circ }$.
- S2) Only non-additive noise ($w_{k-1}^{\prime }=0$): $m_{w}=0$, $\sigma_{v}=0.1$ m/s and $\sigma_{\psi }=0.1^{\circ }$.
- S3) Both additive and non-additive noise with the values in S1 and S2.

In the three scenarios we consider different mismatches $dD=dD_{T}=dD_{B}=0.1,0.5$ and 1 m, and true values $D_{B}=3$ m and $D_{T}=3$ m. We compare five filters:

(i) EKF${}_{ref}$ is the benchmark using the true system ($dD_{T}=dD_{B}=0$).
(ii) EKF stands for the mismatched filter, that is, using the mismatched system input values ${\hat{D}}_{B}$ and ${\hat{D}}_{T}$.
(iii) LCEKF${}_{DT}$ is a linearly constrained filter only accounting for an erroneous value of $D_{T}$ ($a=D_{T}$ in Equation (51)).
(iv) LCEKF${}_{DB}$ is a linearly constrained filter only mitigating the impact of $dD_{B}$ ($a=D_{B}$ in Equation (51)).
(v) LCEKF${}_{DB,DT}$ accounts for both mismatched inputs ($a={\left[D_{B},D_{T}\right]}^{T}$).

### 4.4. Results

**Figure 3:**
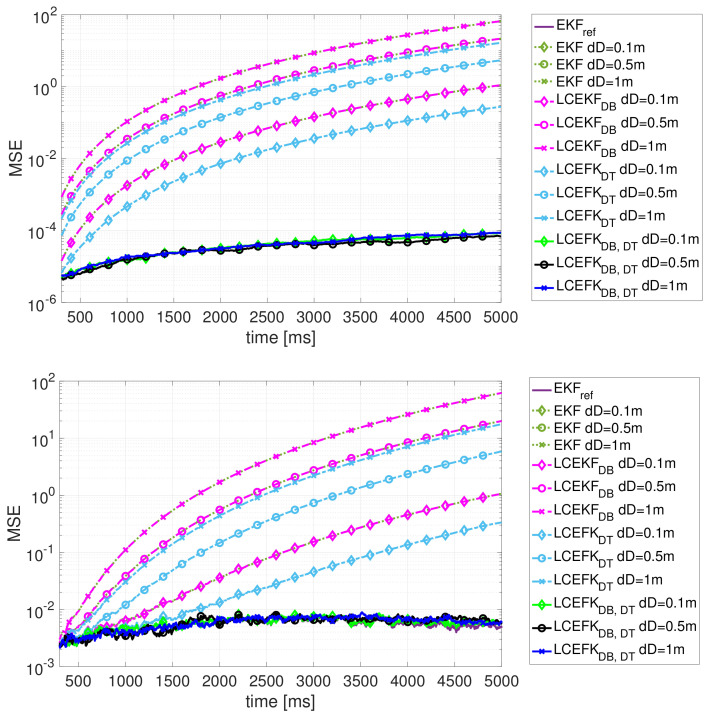

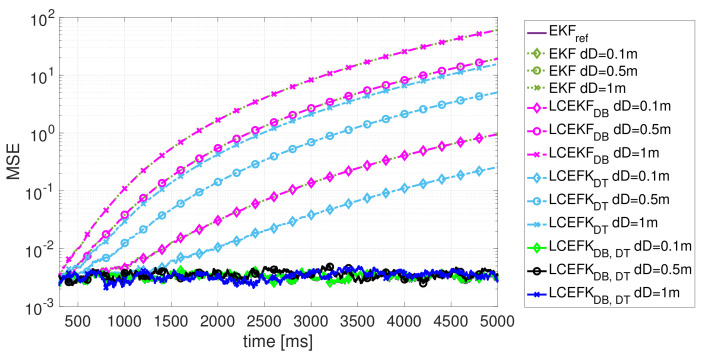
Results for the different sceanrios: (**Top**) Additive noise S1, (**Middle**) non-additive noise S2, and (**Bottom**) both additive and non-additive noise S3. MSE results for the optimal EKF${}_{ref}$, mismatched EKF, and three LCEKF implementations: LCEKF${}_{DB}$ (mitigation of $dD_{B}$), LCEKF${}_{DT}$ (mitigation of $dD_{T}$), and LCEKF${}_{DB,DT}$ (mitigation of both $dD_{B}$ and $dD_{T}$).

Numerical results for the three scenarios are summarised in Figure 3, where we show the position MSE vs time for the five filters obtained over 100 Monte Carlo runs. First, notice that in each scenario (S1, S2 and S3) and regardless of the mismatch value dD the standard EKF always diverges and is not able to correctly estimate the states of interest. That is, even a minor mismatch on the distance from the front wheel to the rear axle induces a performance breakdown on the standard misspecified EKF; therefore, such a mismatch must be accounted for. In addition, the larger the mismatch the faster the performance breakdown. Second, the LCEKF${}_{DB}$ accounting only for a mismatched input on the tracked vehicle ($D_{B}$) does not improve the standard EKF performance (results are superimposed in the plots). Third, the LCEKF${}_{DT}$, which mitigates the tracker input mismatch ($D_{T}$), slightly improves the performance w.r.t. the EKF and LCEKF${}_{dB}$ but does not prevent a performance breakdown. Then, we can conclude that with a mismatch on both vehicles’ distance *D*, and regardless of whether the system is affected by either additive or non-additive noise, either we correctly deal with both mismatched inputs or the filters are not useful anymore.

In contrast to the previous filters, the LCEKF${}_{DB,DT}$, which properly accounts for both input mismatched values, is able to correctly estimate the states and avoid the performance breakdown exhibited by the mismatched filters, regardless of the mismatched value dD, and therefore it is a powerful solution to coping with a possible system model mismatch. Notice that the LCEKF${}_{DB,DT}$ performance is almost superimposed with the optimal EKF${}_{ref}$ in the plots, which again proves the good behaviour of the LCEKF for both additive and non-additive noises. It is worth pointing out that the non-additive noise has a larger impact than the additive one on the system dynamics, which can be seen from the optimal MSE results (i.e., MSE EKF${}_{ref}$$\approx 10^{-4}$ for the additive noise case S1 and MSE EKF${}_{ref}$$\approx 0.5\times 10^{-2}$ for both non-additive noise cases S2 and S3); this drives the estimation error (MSE EKF${}_{ref}$ S2 ≈ MSE EKF${}_{ref}$ S3). This further supports the need for filters able to cope with non-additive noise and system inputs, and the interest of the robust LCEKF formulation proposed in this contribution.

## 5. Conclusions

In this contribution we explored the use of linear constraints to design robust nonlinear KF-type filtering strategies. This was shown in previous contributions to be a promising solution to mitigate parametric modelling errors in both system functions, a problem of interest in real-life applications. In that perspective, we derived a linearly constrained EKF for systems affected by non-additive noise and system inputs, which generalises previous solutions only able to cope with either linear systems or additive noises. Within this framework, it was shown how to exploit linear constraints to mitigate possible nonlinear system model parametric misspecifications on both process and measurement functions. This approach encompasses both errors on system function parameters and system inputs, as well as on noise mean values. A two vehicle robust tracking and navigation problem was used to show the validity and performance improvement provided by the robust LCEKF with respect to state-of-the-art solutions. In addition, it was shown that the non-additive noise has a larger impact than the additive one on the system dynamics, and drives the estimation error, which further supports the need for filters able to cope with non-additive noise and system inputs, and the interest of the robust LCEKF formulation.

As future work, it would be of interest to analyse the use of linear constraints within the context of sigma-point filtering, to better cope with the system nonlinearity and avoid the EKF linearisation errors. Moreover, the proposed solution should be compared to alternative system identification techniques, which, rather than mitigating the impact of mismatched parameters, estimate them together with the states of the system. Another research line would be the generalisation to more general mismatched systems, for instance, considering a possible mismatch on covariance matrices, as well as the comparison or combination with noise statistics’ parameter estimation techniques.

## Appendix A. Details on the Robust Vehicle Tracking and Navigation Example

### Appendix A.1. Tricycle Dynamic Model

The tricycle model (also known as Ackerman model) can be used to model the kinematic behavior of most part of vehicles with three and four wheels. As it is shown in Figure 1, it consists of two main wheels which provide the vehicle speed, and a third wheel which controls the vehicle direction. The turn radius ρ is given by

(A1) $$\rho =\frac{D}{\tan(\psi )}$$

where *D* denotes the distance from the front wheel to the rear axle. We consider the following equations

(A2) $$\begin{array}{c} \left\{\begin{array}{cc} dx & =x^{\prime }-x=S\;\cos\left(\theta +\frac{\Delta \theta }{2}\right)\\ dy & =y^{\prime }-y=S\;\sin\left(\theta +\frac{\Delta \theta }{2}\right)\\ d\theta & =\theta^{\prime }-\theta =L/\rho \end{array}\right., \end{array}$$

where,

(A3) $$\begin{array}{c} \left\{\begin{array}{cc} L & =Vdt\\ \Delta \theta & =L/\rho =\frac{Vdt\;\tan(\psi )}{D}\\ S & =2\rho \;\sin\left(\frac{\Delta \theta }{2}\right)=\frac{2Vdt\;\tan(\psi )}{D}\;\sin\left(\frac{Vdt\;\tan(\psi )}{2\;D}\right)\\ \; & =V\;dt\;\mathrm{sinc}\left(\frac{Vdt\;\tan(\psi )}{2\;D}\right) \end{array}\right., \end{array}$$

and then,

(A4) $$\begin{array}{c} \left\{\begin{array}{cc} dx & =V\;dt\;\mathrm{sinc}\left(\frac{Vdt\;\tan(\psi )}{2\;D}\right)\cos\left(\theta +\frac{Vdt\;\tan(\psi )}{2\;D}\right)\\ dy & =V\;dt\;\mathrm{sinc}\left(\frac{Vdt\;\tan(\psi )}{2\;D}\right)\sin\left(\theta +\frac{Vdt\;\tan(\psi )}{2\;D}\right)\\ d\theta & =\frac{Vdt\;\tan(\psi )}{D} \end{array}\right.. \end{array}$$

### Appendix A.2. Nonlinear Discrete State Model

Considering the state at discrete time *k*, $x_{k}^{T}=\left[{\left(x_{B}\right)}_{k},{\left(y_{B}\right)}_{k},\theta_{k},{\left(x_{T}\right)}_{k},{\left(y_{T}\right)}_{k},\alpha_{k}\right]$ (in the global frame (xOy)), and the previous equations, we can obtain the state equation for the general case with additive and non-additive noise terms, $x_{k}=f_{k-1}\left(x_{k-1},u,w_{k-1}\right)+w_{k-1}^{\prime }$, detailed in Equations (41)–(47). For the three scenarios of interest, we give in the sequel the corresponding approximations (i.e., we omit the dependency with discrete time *k* for the sake of simplicity):

- (S1) Only additive noise ($w_{k-1}=0$, ${\hat{N}}_{k-1}=0$):
  Let
  (A5) $$\begin{array}{cc} q\left(x\right) & =V_{B}\;dt\;tan\left(\phi \right)/\left(2D_{B}\right),g\left(x\right)=\theta +q\left(x\right), \end{array}$$
  (A6) $$\begin{array}{cc} q_{T}\left(x\right) & =V_{T}\;dt\;tan\left(\psi \right)/\left(2D_{T}\right),g_{T}\left(x\right)=\alpha +q_{T}\left(x\right), \end{array}$$
  then
  (A7) $$\begin{array}{cc} \; & {\hat{F}}_{k-1}={\left.\frac{\partial f_{k-1}\left(x_{k-1},u\right)}{\partial x_{k-1}^{T}}\right|}_{x_{k-1}={\hat{x}}_{k-1|k-1},u=\hat{u}},\\ \; & \frac{\partial f(x,u)}{\partial x^{T}}=\left[\begin{array}{cccccc} 1 & 0 & -V_{B}\;dt\;\mathrm{sinc}\left(q\left(x\right)\right)\sin\left(g\left(x\right)\right) & 0 & 0 & 0\\ 0 & 1 & V_{B}\;dt\;\mathrm{sinc}\left(q\left(x\right)\right)\cos\left(g\left(x\right)\right) & 0 & 0 & 0\\ 0 & 0 & 1 & 0 & 0 & 0\\ 0 & 0 & 0 & 1 & 0 & -V_{T}\;dt\;\mathrm{sinc}\left(q_{T}\left(x\right)\right)\sin\left(g_{T}\left(x\right)\right)\\ 0 & 0 & 0 & 0 & 1 & V_{T}\;dt\;\mathrm{sinc}\left(q_{T}\left(x\right)\right)\cos\left(g_{T}\left(x\right)\right)\\ 0 & 0 & 0 & 0 & 0 & 1 \end{array}\right]. \end{array}$$
- (S2) Only non-additive noise ($w_{k-1}^{\prime }=0$):
  Let
  (A8) $$\begin{array}{cc} q\left(x\right) & =V_{B}\;dt\;tan\left(\phi \right)/\left(2D_{B}\right),g\left(x\right)=\theta +q\left(x\right) \end{array}$$
  (A9) $$\begin{array}{cc} q_{T}\left(x\right) & =\left(V_{T}+w_{v}\right)\;dt\;tan\left(\psi +w_{\psi }\right)/\left(2D_{T}\right),g_{T}\left(x\right)=\alpha +q_{T}\left(x\right), \end{array}$$
  then
  (A10) $$\begin{array}{cc} \; & {\hat{F}}_{k-1}={\left.\frac{\partial f_{k-1}\left(x_{k-1},u,w_{k-1}\right)}{\partial x_{k-1}^{T}}\right|}_{x_{k-1}={\hat{x}}_{k-1|k-1},u=\hat{u},w_{k-1}=m_{w}=0}, \end{array}$$
  $$\begin{array}{cc} \; & \frac{\partial f\left(x,u,w\right)}{\delta x^{T}}=\\ \; & \left[\begin{array}{cccccc} 1 & 0 & -V_{B}\;dt\;\mathrm{sinc}\left(q\left(x\right)\right)\sin\left(g\left(x\right)\right) & 0 & 0 & 0\\ 0 & 1 & V_{B}\;dt\;\mathrm{sinc}\left(q\left(x\right)\right)\cos\left(g\left(x\right)\right) & 0 & 0 & 0\\ 0 & 0 & 1 & 0 & 0 & 0\\ 0 & 0 & 0 & 1 & 0 & -\left(V_{T}+w_{v}\right)\;dt\;\mathrm{sinc}\left(q_{T}\left(x\right)\right)\sin\left(g_{T}\left(x\right)\right)\\ 0 & 0 & 0 & 0 & 1 & \left(V_{T}+w_{v}\right)\;dt\;\mathrm{sinc}\left(q_{T}\left(x\right)\right)\cos\left(g_{T}\left(x\right)\right)\\ 0 & 0 & 0 & 0 & 0 & 1 \end{array}\right], \end{array}$$
  (A11) $$\begin{array}{cc} \; & {\hat{N}}_{k-1}={\left.\frac{\partial f_{k-1}\left(x_{k-1},u,w_{k-1}\right)}{\partial w_{k-1}^{T}}\right|}_{x_{k-1}={\hat{x}}_{k-1|k-1},u=\hat{u},w_{k-1}=m_{w}=0}, \end{array}$$
  (A12) $$\begin{array}{cc} \; & \frac{\partial f(x,u,w)}{\partial w^{T}}=\left[\begin{array}{cc} 0 & 0\\ 0 & 0\\ 0 & 0\\ \frac{\partial f_{4}\left(x,u,w\right)}{\partial w_{v}} & \frac{\partial f_{4}\left(x,u,w\right)}{\partial w_{\psi }}\\ \frac{\partial f_{5}\left(x,u,w\right)}{\delta w_{v}} & \frac{\partial f_{5}\left(x,u,w\right)}{\partial w_{\psi }}\\ \frac{\partial f_{6}\left(x,u,w\right)}{\partial w_{v}} & \frac{\partial f_{6}\left(x,u,w\right)}{\partial w_{\psi }} \end{array}\right], \end{array}$$
  where
  $$\begin{array}{cc} \frac{\partial f_{4}\left(x,u,w\right)}{\partial w_{v}} & =dt\;\mathrm{sinc}\left(q_{T}\left(x\right)\right)\cos\left(g_{T}\left(x\right)\right)-dt\;q_{T}\left(x\right)\mathrm{sinc}\left(q_{T}\left(x\right)\right)\sin\left(g_{T}\left(x\right)\right)\\ \; & +dt\;\left(\cos\left(q_{T}\left(x\right)\right)-\mathrm{sinc}\left(q_{T}\left(x\right)\right)\right)\cos\left(g_{T}\left(x\right)\right),\\ \frac{\partial f_{4}\left(x,u,w\right)}{\partial w_{\psi }} & =-\frac{{\left(V_{T}+w_{v}\right)}^{2}\;dt^{2}}{2D_{T}}\left(1+\tan^{2}\left(\psi +w_{\psi }\right)\right)\mathrm{sinc}\left(q_{T}\left(x\right)\right)\sin\left(g_{T}\left(x\right)\right)\\ \; & +\frac{\left(V_{T}+w_{v}\right)\;dt\;\left(1+\tan^{2}\left(\psi +w_{\psi }\right)\right)}{\tan(\psi +w_{\psi })}\cos\left(g_{T}\left(x\right)\right)\cos\left(q_{T}\left(x\right)\right)\\ \; & -\frac{\left(V_{T}+w_{v}\right)\;dt\;\left(1+\tan^{2}\left(\psi +w_{\psi }\right)\right)}{\tan(\psi +w_{\psi })}\cos\left(g_{T}\left(x\right)\right)\sin c\left(q_{T}\left(x\right)\right),\\ \frac{\partial f_{5}\left(x,u,w\right)}{\partial w_{v}} & =dt\;\mathrm{sinc}\left(q_{T}\left(x\right)\right)\sin\left(g_{T}\left(x\right)\right)+dt\;q_{T}\left(x\right)\;\mathrm{sinc}\left(q_{T}\left(x\right)\right)\cos\left(g_{T}\left(x\right)\right)\\ \; & +dt\;\left(\cos\left(q_{T}\left(x\right)\right)-\mathrm{sinc}\left(q_{T}\left(x\right)\right)\right)\sin\left(g_{T}\left(x\right)\right),\\ \frac{\partial f_{5}\left(x,u,w\right)}{\partial w_{\psi }} & =\frac{{\left(V_{T}+w_{v}\right)}^{2}\;dt^{2}}{2D_{T}}\;\left(1+\tan^{2}\left(\psi +w_{\psi }\right)\right)\mathrm{sinc}\left(q_{T}\left(x\right)\right)\cos\left(g_{T}\left(x\right)\right)\\ \; & +\frac{\left(V_{T}+w_{v}\right)\;dt\;\left(1+\tan^{2}\left(\psi +w_{\psi }\right)\right)}{\tan(\psi )}\sin\left(g_{T}\left(x\right)\right)\cos\left(q_{T}\left(x\right)\right)\\ \; & -\frac{\left(V_{T}+w_{v}\right)\;dt\;\left(1+\tan^{2}\left(\psi +w_{\psi }\right)\right)}{\tan(\psi +w_{\psi })}\sin\left(g_{T}\left(x\right)\right)\mathrm{sinc}\left(q_{T}\left(x\right)\right),\\ \frac{\partial f_{6}\left(x,u,w\right)}{\partial w_{v}} & =\frac{dt}{D_{T}}\tan\left(\psi +w_{\psi }\right),\\ \frac{\partial f_{6}\left(x,u,w\right)}{\partial w_{\psi }} & =\frac{(V+w_{v})dt}{D_{T}}\left(1+\tan^{2}\left(\psi +w_{\psi }\right)\right). \end{array}$$
- (S3) Both additive and non-additive noise: in this case ${\hat{F}}_{k-1}$ and ${\hat{N}}_{k-1}$ are computed as in S2)

### Appendix A.3. Nonlinear Measurement Model

The nonlinear measurement model is given in Equation (48). To use the EKF and LCEKF we need to compute the approximation ${\hat{H}}_{k}={\left.\frac{\partial h_{k}\left(x_{k},v_{k},\hat{‘}\right)}{\partial x_{k}^{T}}\right|}_{x_{k}={\hat{x}}_{k|k-1}^{b}}={\left.\frac{\partial h_{k}\left(x_{k}\right)}{\partial x_{k}^{T}}\right|}_{x_{k}={\hat{x}}_{k|k-1}^{b}}$, which is given by

(A13) $${\hat{H}}_{k}={\left.\left[\begin{array}{cccccc} -\cos(\alpha_{k}) & -\sin(\alpha_{k}) & 0 & \cos(\alpha_{k}) & \sin(\alpha_{k}) & \left(\begin{array}{c} -\sin\left(\alpha_{k}\right)\left({\left(x_{T}\right)}_{k}-{\left(x_{B}\right)}_{k}\right)\\ +\cos\left(\alpha_{k}\right)\left(\left(y_{T}\right)-{\left(y_{B}\right)}_{k}\right) \end{array}\right)\\ \sin(\alpha_{k}) & -\cos(\alpha_{k}) & 0 & -\sin(\alpha_{k}) & \cos(\alpha_{k}) & \left(\begin{array}{c} -\cos\left(\alpha_{k}\right)\left(x_{T}-x_{B}\right)\\ -\sin\left(\alpha_{k}\right)\left({\left(y_{T}\right)}_{k}-{\left(y_{B}\right)}_{k}\right) \end{array}\right)\\ 0 & 0 & 1 & 0 & 0 & -1\\ 0 & 0 & 0 & 1 & 0 & 0\\ 0 & 0 & 0 & 0 & 1 & 0\\ 0 & 0 & 0 & 0 & 0 & 1 \end{array}\right]\right|}_{x_{k}={\hat{x}}_{k|k-1}^{b}}.$$

Notice that in this case ${\hat{M}}_{k}=0$.

### Appendix A.4. Linear Constraints

As already stated, the goal is to mitigate the possible mismatch impact by exploiting the constraints

(A14) $$\left(I-L_{k}{\hat{H}}_{k}\right)dF_{k-1}=0,\;{\left.dF_{k}=\frac{\partial f_{k}\left(x_{k},u,w_{k}\right)}{\partial a^{T}}\right|}_{x_{k}={\hat{x}}_{k|k},u=\hat{u},w_{k}=m_{w}=0},$$

with $\hat{u}={\left[V_{B},\phi ,{\hat{D}}_{B},V_{T},\psi ,{\hat{D}}_{T}\right]}^{T}$ and $a={\left[D_{B},D_{T}\right]}^{T}$. Omitting the dependency with discrete time *k* for the sake of simplicity, such constraint is computed as follows. Let

$$\begin{array}{cc} q\left(x\right) & =V_{B}\;dt\;tan\left(\phi \right)/\left(2D_{B}\right),g\left(x\right)=\theta +q\left(x\right),\\ q_{T}\left(x\right) & =\left(V_{T}+w_{v}\right)\;dt\;tan\left(\psi +w_{\psi }\right)/\left(2D_{T}\right),g_{T}\left(x\right)=\alpha +q_{T}\left(x\right), \end{array}$$

(A15) $$\begin{array}{cc} \; & \frac{\partial f(x,u)}{\partial a^{T}}=\left[\begin{array}{cc} \frac{\partial f_{1}\left(x,u\right)}{\partial D_{B}} & \frac{\partial f_{1}\left(x,u\right)}{\partial D_{T}}\\ \frac{\partial f_{2}\left(x,u\right)}{\partial D_{B}} & \frac{\partial f_{2}\left(x,u\right)}{\partial D_{T}}\\ \frac{\partial f_{3}\left(x,u\right)}{\partial D_{B}} & \frac{\partial f_{3}\left(x,u\right)}{\partial D_{T}}\\ \frac{\partial f_{4}\left(x,u\right)}{\partial D_{B}} & \frac{\partial f_{4}\left(x,u\right)}{\partial D_{T}}\\ \frac{\partial f_{5}\left(x,u\right)}{\partial D_{B}} & \frac{\partial f_{5}\left(x,u\right)}{\partial D_{T}}\\ \frac{\partial f_{6}\left(x,u\right)}{\partial D_{B}} & \frac{\partial f_{6}\left(x,u\right)}{\partial D_{T}} \end{array}\right]=\left[\begin{array}{cc} \frac{\partial f_{1}\left(x,u\right)}{\partial D_{B}} & 0\\ \frac{\partial f_{2}\left(x,u\right)}{\partial D_{B}} & 0\\ \frac{\partial f_{3}\left(x,u\right)}{\partial D_{B}} & 0\\ 0 & \frac{\partial f_{4}\left(x,u\right)}{\partial D_{T}}\\ 0 & \frac{\partial f_{5}\left(x,u\right)}{\partial D_{T}}\\ 0 & \frac{\partial f_{6}\left(x,u\right)}{\partial D_{T}} \end{array}\right], \end{array}$$

$$\begin{array}{cc} \frac{\partial f_{1}\left(x,u\right)}{\partial D_{B}} & =\frac{V_{B}\;dt}{D_{B}}\mathrm{sinc}\left(q\left(x\right)\right)\cos\left(g\left(x\right)\right)-\frac{V_{B}\;dt}{D_{B}}\cos\left(q\left(x\right)\right)\cos\left(g\left(x\right)\right)\\ \; & +\frac{V_{B}\;dt}{D_{B}}q\left(x\right)\mathrm{sinc}\left(q\left(x\right)\right)\sin\left(g\left(x\right)\right),\\ \frac{\partial f_{2}\left(x,u\right)}{\partial D_{B}} & =-\frac{V_{B}\;dt}{D_{B}}\mathrm{sinc}\left(q\left(x\right)\right)\cos\left(g\left(x\right)\right)-\frac{V_{B}\;dt}{D_{B}}\cos\left(q\left(x\right)\right)\sin\left(g\left(x\right)\right)\\ \; & -\frac{V_{B}\;dt}{D_{B}}q\left(x\right)\mathrm{sinc}\left(q\left(x\right)\right)\cos\left(g\left(x\right)\right),\\ \frac{\partial f_{3}\left(x,u\right)}{\partial D_{B}} & =\frac{2q\left(x\right)}{D_{B}},\\ \frac{\partial f_{4}\left(x,u\right)}{\partial D_{T}} & =\frac{V_{T}\;dt}{D_{T}}\mathrm{sinc}\left(q_{T}\left(x\right)\right)\cos\left(g_{T}\left(x\right)\right)-\frac{V_{T}\;dt}{D_{T}}\cos\left(q_{T}\left(x\right)\right)\cos\left(g_{T}\left(x\right)\right)\\ \; & +\frac{V_{T}\;dt}{D_{T}}q_{T}\left(x\right)\mathrm{sinc}\left(q_{T}\left(x\right)\right)\sin\left(g_{T}\left(x\right)\right),\\ \frac{\partial f_{5}\left(x,u\right)}{\partial D_{T}} & =-\frac{V_{T}\;dt}{D_{T}}\mathrm{sinc}\left(q_{T}\left(x\right)\right)\sin\left(g_{T}\left(x\right)\right)-\frac{V_{T}\;dt}{D_{T}}\cos\left(q_{T}\left(x\right)\right)\sin\left(g_{T}\left(x\right)\right)\\ \; & -\frac{V_{T}\;dt}{D_{T}}q_{T}\left(x\right)\mathrm{sinc}\left(q_{T}\left(x\right)\right)\cos\left(g_{T}\left(x\right)\right),\\ \frac{\partial f_{6}\left(x,u\right)}{\partial D_{T}} & =\frac{2q_{T}\left(x\right)}{D_{T}}. \end{array}$$
